# Lactobacilli and human dental caries: more than mechanical retention

**DOI:** 10.1099/mic.0.001196

**Published:** 2022-06-07

**Authors:** Zezhang T. Wen, Xiaochang Huang, Kassapa Ellepola, Sumei Liao, Yihong Li

**Affiliations:** ^1^​ Department of Prosthodontics, School of Dentistry and Department of Microbiology, Immunology and Parasitology, School of Medicine, Louisiana State University Health Sciences Center, New Orleans, LA, USA; ^2^​ Department of Public and Ecosystem Health, College of Veterinary Medicine, Cornel University, Ithaca, NY, USA; ^†^​ Present address: Analysis and Testing Center, Nanchang University, 235 Nanjing East Load, Qingshan Lake District, Nanchang, PR China; ^‡^​ Present address: Department of Oral Biology, College of Dentistry, University of Illinois Chicago, Chicago, IL, USA

**Keywords:** dental caries, lactobacillus, *Streptococcus mutans*, biofilm formation, oral microecology, genetic regulation

## Abstract

Lactobacilli have been considered as major contributors to human dental caries for over a century. Recent *in vitro* model studies have shown that when compared to *Streptococcus mutans,* a keystone pathogen of human dental caries, the ability of lactobacilli to form biofilms is poor, although differences exist between the different major species. Further studies using molecular and bioinformatics approaches provide evidence that multiple mechanisms, including adhesin-receptor mediated physical contact with *S. mutans *, facilitate the adherence and establishment of lactobacilli on the tooth surface. There is also evidence that under conditions like continuous sugar consumption, weak acids and other antimicrobials such as bacteriocins from lactobacilli can become detrimental to the microbial community, especially those in the proximity. Details on the underlying mechanisms of how different *Lactobacillus* sp. establish and persist in the highly complex microbiota on the tooth surface await further investigation.

## Introduction

Lactobacilli were the first micro-organisms implicated in human dental caries over a century ago and was the main etiological candidate before the mutans streptococci became dominant in the literature in the 1950s [1, 2]. From clinical isolation and cultivation to 16S rRNA-based analysis and more recently, deep sequencing of the plaque microbiota, *Lactobacillus* sp. are frequently identified at active carious sites, especially in lesions with advanced caries in adult and paediatric patients [3–6]. However, unlike *Streptococcus mutans *, a keystone cariogenic bacterium whose pathophysiology and virulence attributes have been well characterized through extensive investigations in recent decades, major knowledge gaps remain concerning the role of *Lactobacillus* sp. and especially, the mechanisms how they establish and persist on the tooth surface and facilitate the development of carious lesions.

Dental caries is a clinic manifestation of continued demineralization of the tooth enamel resulting from acids produced from sugar fermentation by bacteria in the plaque microbiota on the tooth surface, under the gum and in the proximal surfaces. Recent advances, including data from the human oral microbiome project (www.homd.org), have clearly demonstrated that dental caries results from a dysbiotic plaque microbiota in response to host and environmental perturbations that include saliva deficiency, poor oral hygiene and continuous consumption of fermentable sugars. A cariogenic plaque microbiota is featured with disproportional increases of highly acidogenic and aciduric species, which include mutans streptococci, lactobacilli and bifidobacteria. It is well-known that the abilities to colonize and persist on the tooth surface, to catabolize sugars and produce weak acidic metabolites, and to tolerate the acids and the resulting low pH environment are traits considered essential for a bacterium to cause carious lesions [7]. Many studies using *in vivo* models, mostly prior to the mutans streptococci paradigm, have examined the roles of lactobacilli in dental caries [8–11], but questions remain inexplicit on the molecular mechanisms concerning how *Lactobacillus* sp. colonize and establish on the tooth surface and how they interact with the micro-environment and with other major bacterial species in the plaque microbiota, influencing the development including temporal structure and composition of the plaque microbiota. On the other hand, many studies have been done with the food and probiotic lactobacilli, and several articles have recently offered comprehensive reviews on these lactobacilli including comparative genomics and the potential of biotechnological engineering [12–18]. This review attempts to highlight recent studies on oral lactobacilli with a focus on bacterial biofilm formation and its regulation including the roles of inter-species interactions.

## Major *Lactobacillus* species in dental caries

From over a century of extensive investigations, the connection of lactobacilli to human dental caries in children and adults has been well established [1, 5, 6]. Lactobacilli are among the micro-organisms that can be found in the oral cavity soon after babies are born. The lactobacilli in the oral cavity of infants are believed to mainly come from the mouths of their parents, especially their mothers due to close contacts, and from the foods they eat, including milk and different fermented products. There is currently no effective means to determine the definite origin of or differentiate the types of *Lactobacillus* sp. In the oral cavity, lactobacilli can be found mostly in saliva, on the surface of the mucosa, the hard palate and the dorsum of the tongue. They are mostly thought to be transient in the mouth and their numbers on the tooth surfaces of healthy infants and young children are usually low [5, 6, 19]. Low affinity to the tooth surface is thought to be the major factor that contributes to its low prevalence in the plaque microbiota of healthy individuals [8, 10]. However, lactobacilli tend to increase in numbers with increasing intake of fermentable sugars in the diet, a situation that is also conducive to dental caries. In individuals with active dental caries, both the diversity and prevalence of lactobacilli in dental plaque, especially the active carious sites, increase significantly, as compared to individuals who are caries-free [5, 6]. Major differences in diversity and prevalence among different studies also exist, which can be at least in part attributed to the differences in study populations, severity of the disease at the time of the study, and the methodologies used, notably cultures versus DNA-based methods [20]. Among the *Lactobacillus* sp. identified from carious lesions, the most dominant species in adult and childhood dental caries, including severe early childhood caries, are *L. casei/paracasei, L. fermentum, L. rhamnosus, L. gasseri, L. salivarius* and *L. plantarum* [5, 6] (Table S1, available in the online version of this article). Those with low prevalence were thought to be more likely transient contaminants from food or other sources with no major role in dental caries. A recent study of severe early childhood caries by Caufield *et al*. [5] also showed that most of the *Lactobacillus* sp. existed as cohabitants with other lactobacilli. A few species, including *L. casei/paracasei, L. fermentum* and *L. salivarius,* were identified as the single occupant of the caries lesions. It is unclear if any of these three major species are more pathogenic than the others in terms of their cariogenesis. For more information on the natural history of the lactobacilli in the oral cavity and their association to dental caries, consult recent reviews by Caufield *et al.* and Duar *et al.* [5, 21].

## Sugar fermentation and acid production

Lactobacilli have been widely used as starter cultures in food preservation and production, and some are considered as probiotic conferring health benefits on the host upon consumption/application. For various reasons, these lactobacilli have been extensively studied concerning their bacterial physiology, especially carbohydrate metabolism and regulation and the potential of biotechnological engineering. Lactobacilli are strictly fermentative bacteria, known for their high capacity to utilize a variety of carbohydrates. In fact, lactobacilli were originally grouped taxonomically according to their major carbohydrate utilization patterns as homofermentative, facultatively heterofermentative, and obligate heterofermentative lactobacilli [19]. Homofermentative lactobacilli ferment hexoses with lactic acid as the primary by-products, while heterofermentative lactobacilli produce lactic acid, ethanol, acetic acid and carbon dioxide as end-products. *Lactobacillus* sp. are diverse, and major differences also exist within the different species (Table S1), as revealed by 16S rRNA and whole-genome sequence analyses [13, 14, 22, 23]. However, they all possess a broad repertoire of enzymes that enable them to utilize various carbohydrates, which are rich in oligosaccharides and starches in the oral cavity. To name a few, they include glucosidases, fructosidases, galactosidases, glucansucrase, levansucrases and fructansucrases for oligosaccharides and the α- and β-amylases and amyloglucosidases for starches [13, 22–27]. Different from dairy isolates, clinical and oral *L. rhamnosus* were found to possess l-fucisidase, which enables them to utilize various fucosyl-glycoconjugates on epithelia cell surface and intestinal mucin [22]. Lactobacilli possess rich and redundant transport systems for a variety of mono- and disaccharides, and to a less degree, trisaccharides, which include the high-affinity, sugar-specific phosphotransferase transporter systems (PTSs). Like many other bacteria, some of the PTSs possess typical enzyme II with complete subunits, while others lack one or more of the components (incomplete PTSs) [5, 13, 24]. The number of the PTSs varies significantly between different *Lactobacillus* sp. (Table S1) [23]. For instance, *L. rhamnosus* 1.0320 has the most at 51 (with 33 complete PTSs and 18 incomplete PTSs) (Huang and Wen, personal communication), while the least was found in heterofermenters *L. brevis* ATCC 367 (five incomplete PTSs only) and *L. reuteri* F275 (four incomplete PTSs only) [28]. Besides, lactobacilli also contain non-sugar-specific ABC transporters that the bacteria utilize to take up different substrates in the expense of ATP [28, 29]. For example, *L. acidophilus* possesses a four-gene operon encoding a LacI-type regulator, a four-component ABC transporter, a fructosidase and a sucrose phosphorylase, respectively, which was found to function similarly as the multiple sugar metabolism (MSM) system of *S. mutans* [30]. Lactobacilli also possess diverse groups of glucosyltransferases (Gtfs) [31–34], including glycogen synthase and glycogen phosphorylase for glycogen biosynthesis [13]. Glycogen is one of the major carbohydrate storage mechanisms for lactobacilli, a capacity believed to allow them advantage in competition over others [35]. Lactobacilli also produce extracellular polysaccharides (EPS) that are mostly α [1, 4]-linked glucose polymers and some with α [1, 6] linkages [22, 31]. Recent studies by Caufield *et al.* showed that about half of the *L. fermentum* analysed possesses the genes for the extracellular glucans, but they were remarkably absent in other species associated with severe childhood caries [5]. No evidence suggests that lactobacilli synthesize any major adhesive EPS from sucrose [8, 36]*,* which in *S. mutans* is predominantly α [3,1]-linked and known to play a central role in its adherence and cariogenicity [37].

Carbohydrate metabolism in lactobacilli is highly regulated by substrate induction and catabolite repression in response to substrate availability and presence of other more readily metabolizable carbohydrate sources [29, 38]. In addition, environmental conditions, such as oxygen tension, temperature and probably presence of other bacterial species, can also have an impact on the regulation of carbohydrate metabolism. Like *S. mutans,* the carbon catabolite repression protein (CcpA) in lactobacilli is a multi-functional regulator that plays a central role in regulation of carbohydrate metabolism, including modulation of the fermentation pathways from homolactic fermentation to heterofermentation in response to various environmental conditions including sugar source and availability and aeration [39–44]. There is evidence that certain species of lactobacilli displayed distinctive metabolomics profiles when they were grown together with *S. mutans,* when compared to the respective mono-species cultures [45], although details on the mechanisms that regulate the metabolic pathways remain largely unknown.

## Acid tolerance and acid-tolerance responses

**Fig. 1.**
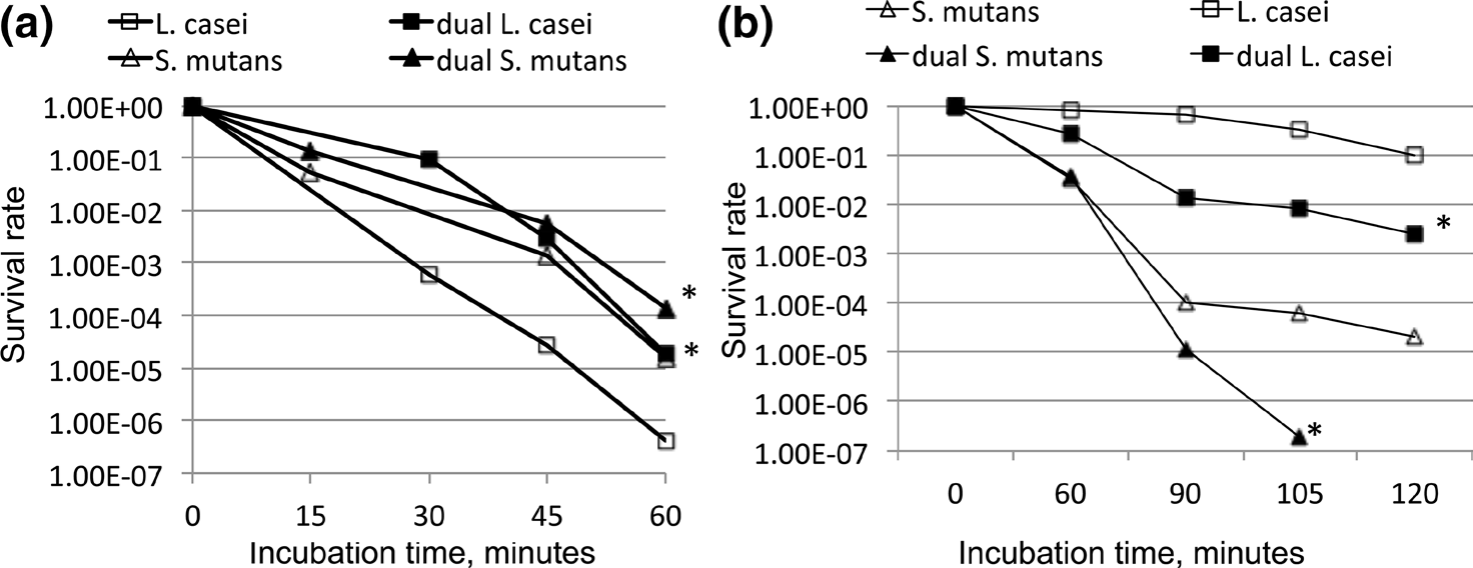
*
S. mutans* and *L. casei* in dual-species cultures displayed altered survival rates as compared to those grown alone, when subjected to acid- (a) and hydrogen-peroxide- (b) killing assays. For acid-killing assays, *L. casei* was incubated in 0.1 M glycine buffer, pH 2.0 for periods as indicated, and *S. mutans* was incubated in buffer of pH 2.8 for the same periods. For hydrogen-peroxide-killing assays, *S. mutans* and *L. casei* were incubated in glycine buffer containing 0.2% hydrogen peroxide. *, *P*<0.001 vs the respective mono-species cultures. (From Wen *et al.*, 2017. *Front. Cell. Infect. Microbiol.* 7 : 524. doi: 10.3389/fcimb.2017.00524).

Lactobacilli are well known for their ability to rapidly break down sugars to acidic end-products, of which at least half is lactic acid. Consequently, the pH value of their immediate environment can quickly plummet to levels where the metabolic processes including glycolysis and bacterial cell growth become inhibited and even completely stopped, although differences in culture pH and the ability to cope with the environmental pH exist among different species [45–47]. Since the work of Stephan and Hemmens [48], numerous studies have shown not only the acidogenic capacities of lactobacilli but also their ability to tolerate weak acids and low pH environment [45, 49–51]. Comparing to *S. mutans* and members of the mutans streptococci*,* lactobacilli were shown to be capable of growing at pH 4.0 [52]. As illustrated using the acid-killing assays*, L. casei* is also significantly more acid tolerant than *S. mutans* (Fig. 1) [45]. Unlike *S. mutans* that reduced its survival rate by >5 logs after 60 min at pH 2.8, *L. casei* survived the same pH for a long period of time. It has been suggested that lactobacilli possess multiple mechanisms to cope with the acid stresses, including passive acid efflux via the cell membrane, active proton pump via F_1_F_0_-ATPase, sodium-proton antiporters, and various alkali production pathways such as the glutaminase and the arginine deiminase, whose ammonia product can neutralize protons [24, 47] (Table S1). In addition, several lactobacilli also have the glutamate decarboxylase (GAD) system, one of the essential acid tolerance systems in many Gram-negative and Gram-positive bacteria [53–55], consisting of the GAD and a glutamate/gamma-aminobutyric acid (GABA) antiporter. With the GAD system, GAD catalyses the decarboxylation of glutamate to produce GABA. The antiport of glutamate and GABA consumes protons elevating intracellular pH and contributing to a proton motive force for ATP synthesis.

Lactobacilli are also capable of launching adaptive acid-tolerance response, which is featured with enhanced acid tolerance, following initial exposure to a low pH condition [45, 56] (Fig. 1). Typically, when compared to cultures grown in medium with buffered pH, cultures grown in regular medium will have a reduced culture pH and a significantly enhanced survival rate when subjected to acid-killing assays. Adaptive acid-tolerance responses often result from elevated expression and activity of the F_1_F_0_-ATPase, molecular chaperones such as DnaK and GroEL, enzymes such as Clp protease, excinuclease, involved in repair and protection of DNA and proteins, and enzymes such as squalene synthase involved in membrane biosynthesis and thus acid efflux [47, 57–59].

Various factors have been shown to play a role in regulation of acid-tolerance response, including GadR, GlnR and two-component signal transduction systems [60–62]. GadR in *L. brevis* functions as a positive regulator of GAD and glutamate/GABA antiporter, thus the GABA conversion from glutamate, and is expressed at a much higher level in the strains isolated from acidic habitats [61]. Besides, nitrogen regulator GlnR is also found to play a role in glutamate-dependent acid resistance by modulating GABA conversion from glutamate [62]. In *L. elbrueckii* subsp. *bulgaricus*, the two-component signal-transduction system HPK1/RR1 was shown to regulate the acid adaptation ability of the bacterium by means of many pathways, including the proton pump-related protein, classical stress-shock proteins, carbohydrate metabolism, nucleotide biosynthesis, DNA repair, transcription and translation, peptide transport and degradation, and cell-wall biosynthesis, etc [58, 60, 63, 64].

## Biofilm formation and regulation

From early studies of human volunteers, selected lactobacilli were found to possess low affinity for the tooth surface [10]. It was found that mechanical retention either directly or indirectly by incorporation of bacterial cells in food remnants play an important role in lactobacilli establishment; and that the presence of teeth and especially, carious lesions may be required for lactobacilli to establish and maintain on the tooth surface in significant numbers [10]. In a recent article, Caufield *et al.* also postulated that a ‘retentive niche’, which includes food remnants and early plaque biofilms and provides physical trapping, retainment and containment, is required for sustained colonization [5].

Many of the *Lactobacillus* strains isolated from dental plaque, comprising different major species, were examined using animal models, including a gnotobiotic rat model, and were found to be moderately or highly cariogenic when the animals were fed with a diet rich in sucrose, but not when fed with glucose and/or starch [8, 9, 11, 65]. However, the level of carious lesions in animals infected with these lactobacilli strains was significantly lower, when compared to those infected with *S. mutans* [11].

**Fig. 2.**
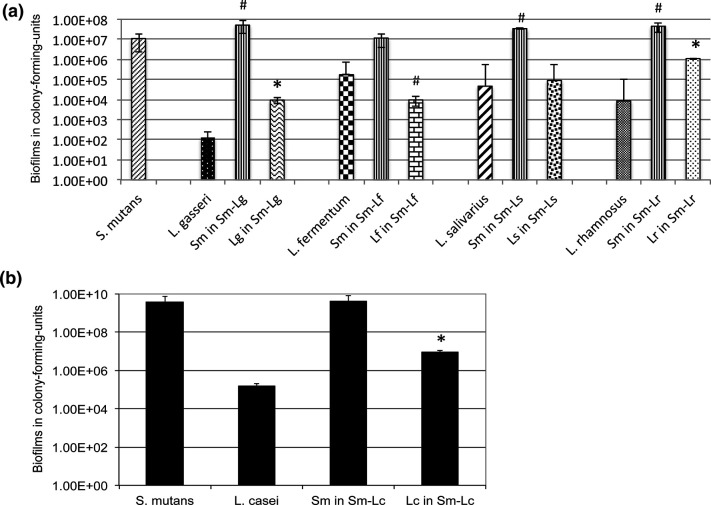
Lactobacilli biofilms when grown alone and together with *S. mutans* under static (a) and continuous flowing (b) conditions. (a) shows 48 h biofilms of *L. gasseri* (Lg), *L. fermentum* (Lf), *L. salivarius* (Ls) and *L. rhamnosus* (Lr) in mono-species and dual-species with *S. mutans* (Sm) grown on glass slides vertically deposited in 50 ml Falcon tubes, with * and # indicating statistical difference at *P*<0.001 and 0.05, respectively, when compared to the respective mono-species biofilms. (b) shows 5 day biofilms of *S. mutans* (Sm) and *L. casei* (Lc) in mono- and dual-species model grown on glass slides in a drip flow biofilm reactor, with * indicating statistical difference at *P*<0.001 relative to its mono-species biofilms. (From Wen *et al., 2017.* Front. Cell. Infect. Microbiol. 7 : 524. doi: 10.3389/fcimb.2017.00524).

*In vitro* model studies of different lactobacilli species, mostly laboratory strains, have also showed that lactobacilli form biofilms poorly, but differences also exist between different species [45, 66]. Using an artificial mouth system, Filoche *et al.* [67] found that biofilm formation by *L. rhamnosus* and *L. plantarum* was poor. In 96-well plates, Wen *et al.* showed that of the most prevalent species analysed, all showed limited ability to form biofilms, although differences exist between the major species tested, with *L. fermentum* forming the best biofilms while the least being detected with *L. gasseri* (Fig. 2) [45, 66]. Differences in growth rates and final culture optical densities were also observed when using different carbohydrate sources and culture media, and improvement in growth was observed when a higher amount of sucrose was supplied in the culture medium, which is consistent with early animal model studies [8, 9]. Unlike *S. mutans,* however, none of the tested *Lactobacillus* strains displayed any major increases in biofilm formation, when grown in sucrose-containing medium as compared to that without; and none produced any water insoluble, adhesive EPS as the *S. mutans* did when grown under similar conditions [8, 9, 36].

Biofilm formation is initiated by bacterial cell-surface interactions, and the cell-surface structure plays a vital role in colonization and persistence. Much of the current knowledge on lactobacilli cell-surface interactions is attributed to the studies of probiotic *Lactobacillus* isolates of the gut. Various components on the cell envelop are implicated as mediators of bacterial adherence, which include cell-wall-associated polysaccharides and teichoic acids and lipoteichoic acids, surface-layer proteins (S-layers), surface-associated adhesins, and multi-functional moonlighting proteins such as GroEL, enolase and collagen-binding proteins [68]. Many *Lactobacillus* sp., but not all, have a S-layer, which is a layer of two-dimensional crystalline of repeating proteinaceous subunits that covers completely the outermost surface of the cell envelop and is responsible for the surface hydrophobicity [69]. In a number of studies, the loss of S-layer has been shown to result in reduced bacterial adherence to different surfaces, although the direct role of the S-layer proteins in bacterial adherence and biofilm formation remains unclear. Lactobacilli also possess LPXTG proteins, which are anchored to the cell envelop by sortase enzymes, including the serine-rich adhesins, although the number of these anchored proteins differ between different species [13, 70–72]. Lactobacilli serine-rich proteins, such as SRRP in probiotic *L. reuteri* [70], share similarities with and differences from the counterparts in other Gram-positive bacteria such as Fap1 of *S. parasanguinis* and GspB of *S. gordonii* whose roles in bacterial adherence and biofilm formation have been well documented. The LPXTG proteins in some probiotic lactobacilli have been shown to play an important role in the interactions with their habitat, including mucus-binding and the associated microbial communities. Certain *Lactobacillus* sp., including *L. rhamnosus* but not *L. casei *, are also piliated, and the pilins are thought to be involved in mucin-binding and inter-cellular signalling [13, 73, 74]. However, currently no further information is available concerning the roles of such proteinaceous fibres in bacterial colonization and biofilm formation. Like gut lactobacilli [68], many *Lactobacillus* sp. from the oral cavity, such as *L. casei/paracasei, L. gasseri, L. rhamnosus, L. fermentum, L. salivarius, L. oris* and *L. vaginalis* strains isolated from patients with severe early-childhood caries*,* also contain genes encoding binding proteins to type I collagens [5], which become exposed during the dentinal caries progression. Consistently, majority of *Lactobacillus* strains tested have showed ability to adhere to surfaces coated with type I collagen, and such adherence can be competitively inhibited with collagen [75], which is consistent with a role of *Lactobacillus* sp. in root and/or coronal caries. This is in contrast with their cariogenic partner *S. mutans,* of which only about 17 % of the isolates contain the *cnm* and *cbm* genes encoding collagen-binding protein [76].

## Inter-species interactions on Lactobacilli biofilm formation

Emerging data suggest that inter-species interactions in the plaque microbiota are central drivers of homeostasis and dysbiosis and thus are the critical determinants of oral health and disease development [77, 78]. Multiple mechanisms function in interactions between different species in the oral microbiota, including synergistic mechanisms such as adhesin-receptor-mediated physical contact and cross-feeding on nutrient utilization [78]. It is well documented that in *in vitro* models, some *Lactobacillus* sp. can drastically increase biofilm formation when co-cultivated with *Actinomyces* sp. and *S. mutans* [45, 66, 67]. When co-cultivated in an artificial mouth system with *Actinomyces naeslundii* and *A. gerencseriae, L. rhamnosus* increased biofilms by as many as 20 times and *L. plantarum* biofilms increased by up to sevenfold, when compared to their respective mono-culture biofilm [67]. In 96-well culture plates, biofilm formation by *L. casei, L. gasseri* and *L. rhamnosus* was increased by almost 2-logs after 48 h when growing in a dual-species model with *S. mutans* [45, 66] (Fig. 2). From recent studies by Wen *et al.* [45] and Huang *et al.* [36], the impact of inter-species interactions on biofilm formation appears to be more species-specific. In contrast, *L. fermentum* in co-cultivation with *S. mutans* decreased biofilms by >tenfold, while no significant differences were measured with *L. salivarius* ssp. *salivarius* [36, 45, 66]. Similar results were obtained on hydroxylapatite discs and glass slides, two commonly used *in vitro* tooth models, and when grown under continuous flowing conditions [45] (Fig. 2b). However, no such effects were measured when they grew with *Veillonella parvula* and *V. dispar* [45, 67].

**Fig. 3.**
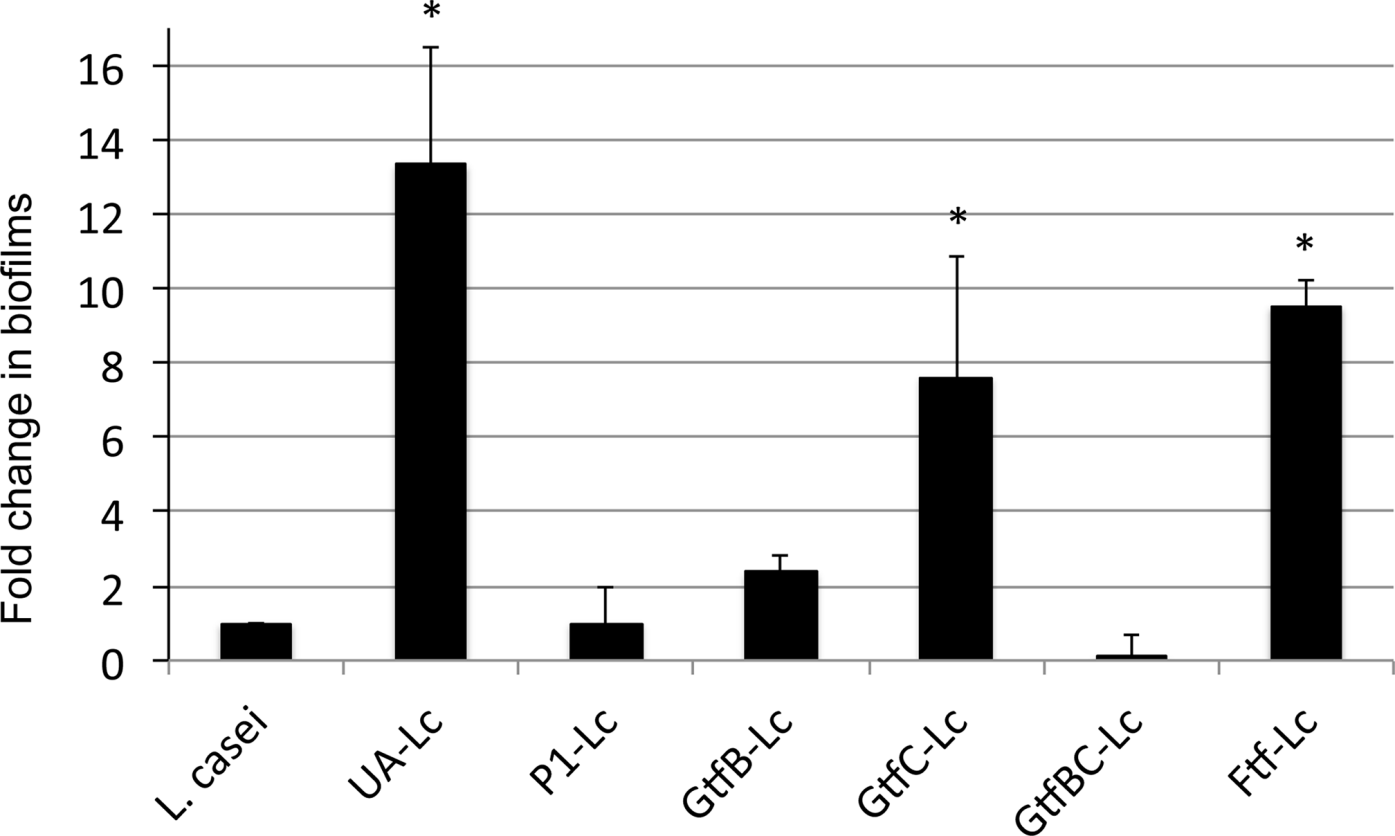
*
L. casei* biofilm formation with *S. mutans* mutants. *L. casei* (Lc) was grown alone or together with *S. mutans* wild-type UA159 (UA) and its mutants deficient of P1, GtfB, GtfC, GtfBC or Ftf on glass slides for 48 h. Data are expressed as the ratio of *L. casei* biofilms (in colony-forming-units) in dual-species over those in mono-species, with * indicating significant differences at *P*<0.001. (From Wen *et al., 2017.* Front. Cell. Infect. Microbiol. 7 : 524. doi: 10.3389/fcimb.2017.00524).

**Fig. 4.**
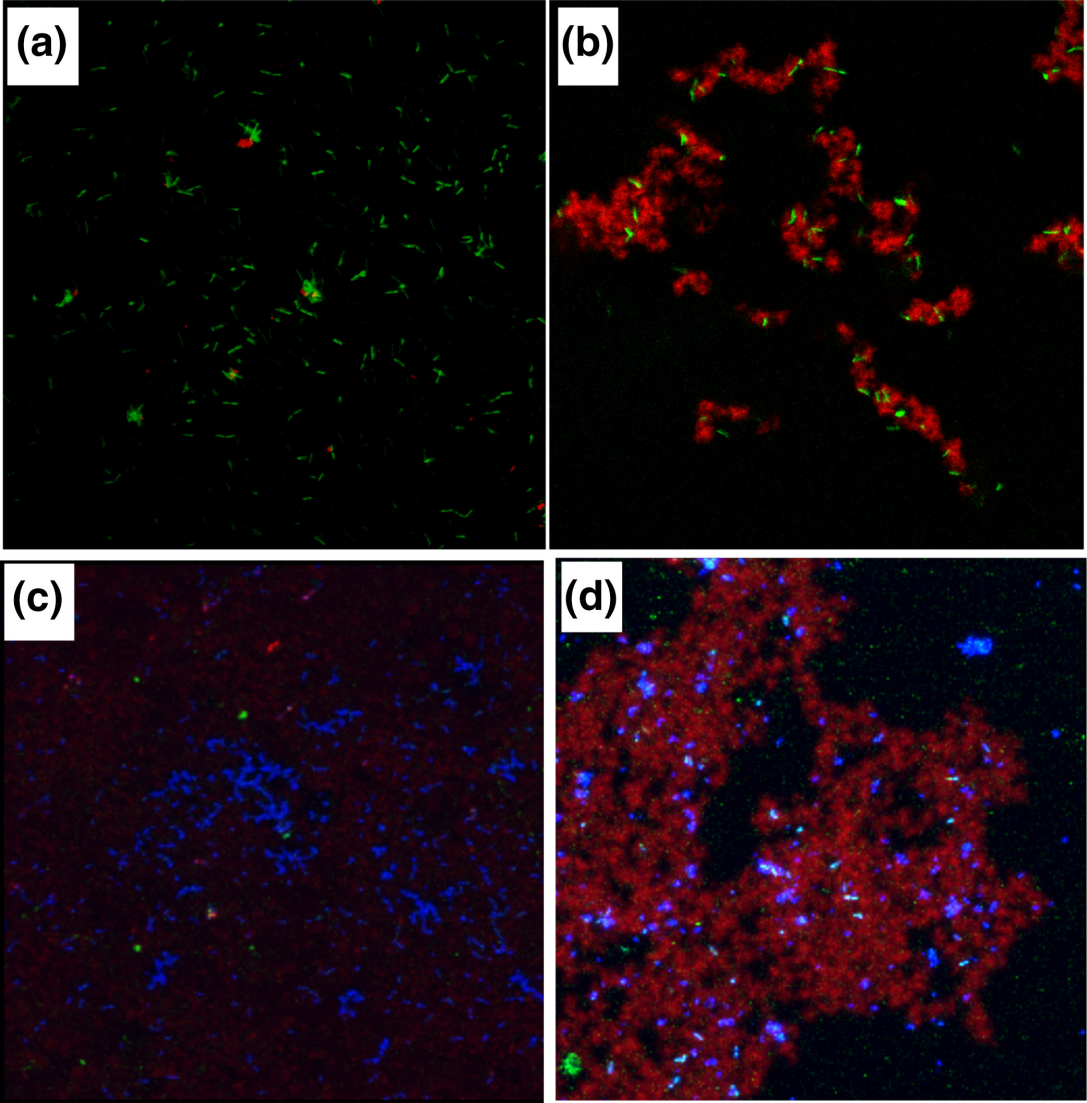
Visualization of glucans synthesized *in situ* by GtfB adsorbed on *L. casei* and *S. mutans* binding to *L. casei* with and without glucans. *L. casei* was incubated with GtfB or buffer, and following washes, exposed to sucrose for 1 h, and glucans were imaged using a confocal microscope. (a) *L. casei* (in green) with GtfB in buffer alone; (b) *L. casei* with bound-GtfB incubated with sucrose showing rich glucans (in red) engulfed in green *L. casei* cells. (c) *S. mutans* (in blue) incubated with green *L. casei* with no glucans on surface; and (d) *S. mutans* (in blue) incubated with *L. casei* coated with GtfB glucans (in red). Images were obtained using an upright Olympus confocal microscope with a 100 x oil objective. (From Wen *et al., 2017.* Front. Cell. Infect. Microbiol. 7 : 524. doi: 10.3389/fcimb.2017.00524).

In an effort to uncover the mechanisms underlying the enhanced biofilm formation, Liao *et al.* showed that deficiency of multi-functional adhesin P1 and glycosyltransferase B (GtfB) in *S. mutans* almost completely demolished the ability of *S. mutans* to facilitate *L. casei* biofilm formation [45] (Fig. 3). GtfB is known for its ability to synthesize adhesive glucans from sucrose, but it can also function as an adhesin and directly bind to other bacteria such as *Candida albicans* [79]. In an *in vitro* adherence assay, purified native GtfB protein was able to bind to *L. casei* cells*,* and when the GtfB-bound bacterial cells were incubated with sucrose, synthesized adhesive glucose polymers that were able to bind to *S. mutans* (Fig. 4). In support of the mechanical retention theory [5, 10], the results further suggest that biofilms of the early colonizers and especially, their extracellular polymers, which include the rich polysaccharides and deoxyribonucleic acids [80, 81], play an important role as a scaffold in the establishment and retention of *Lactobacillus* sp. Besides, the results also provided evidence that other factors including the intercellular interactions mediated by adhesins such as P1 and GtfB and their receptors also actively facilitate the adherence of *Lactobacillus* sp. and the development of inter-species multi-cellular clusters. Further studies on the adhesins and the binding ligands in *S. mutans–Lactobacillus* interaction will shed new light on whether such intercellular interactions are species-specific.

It has been known that a synergistic relationship exists between *L. bulgaricus* and *S. thermophilus* that is featured with stimulated growth and acidification of both during milk fermentation. When grown in a mixed-species consortium in rich medium (*i.e.* brain heart infusion), both *Lactobacillus sp*. and *S. mutans* showed altered behaviours, including enhanced acid production and acid tolerance responses [45]. When co-cultivated with *S. mutans,* all *Lactobacillus* species, except *L. fermentum *, had a reduced cultural pH than the respective mono-species cultures after 24 h. Metabolomics analysis showed that selected metabolites such as succinic acid in the spent culture medium of the *L. casei-S. mutans* dual-species cultures were significantly different from those in both *S. mutans* and *L. casei* mono-species cultures [45]. There is also evidence that growth in dual-species with *S. mutans* led to alterations in metabolic dynamics including changes in amino acid biosynthesis and carbohydrate metabolism [36]. However, currently there is no definitive information concerning how *Lactobacillus sp.* in a mixed-species community alter their metabolic dynamics.

*L. casei* in dual-species with *S. mutans* also displays a drastic enhancement in resistance to acid and low pH, as compared to its mono-species cultures. When incubated in a glycine buffer of pH 2.0, 0.1 M, the survival rate of the mono-species cultures reduced by >3 log after 30 min. In contrast, the survival rate of those grown in the dual-species cultures with *S. mutans* reduced by only 1-log when analysed under the same conditions. Similar trends, although in a much less degree, were also observed with *S. mutans,* which is known for its ability to launch adaptive acid tolerance responses [57]. However, it is currently unclear how such physiological characteristics may affect each other and ultimately the composition and the cariogenicity of the plaque microbiota.

## Probiotic Lactobacilli and their potential in oral health

Lactobacilli are known to produce a variety of compounds including acids and bacteriocins that exert direct antimicrobial activities toward competing bacteria in a community [82] (Table S1). Food-borne lactobacilli have long been utilized for food preservation through production of weak acids and bacteriocins. Probiotic lactobacilli are widely used either alone or with other probiotic species for various health benefits including displacement of putrefactive organisms and restoration of microecological homeostasis. Several *Lactobacillus* species, including *L. salivarius, L. fermentum* and *L. paracasei,* have also been studied for their potential against cariogenic bacteria like *S. mutans* and dental caries [83–90], although the idea of lactobacilli being beneficial from dental cariogenesis point of view remains controversial. Nevertheless, antimicrobial effects against mutans streptococci and some other oral bacteria in both *in vitro* and *in vivo* models are well documented [8, 11, 67, 83–85, 88, 90, 91]. Such antagonistic effects have been measured with reduction of bacterial adherence, glucan production, and biofilm formation *in vitro* and by reduction of caries and caries severity *in vivo*.

When compared to the mono-species cultures, *S. mutans* in dual-species model with *L. casei* under aerobic conditions also displayed a dramatic reduction in survival rate against hydrogen peroxide challenge and consistently, had an altered transcription profile [45, 66]. Among the down-regulated genes are the ones for GtfB and alternative sigma factor ComX, which regulates competence development, bacteriocin production and biofilm formation, while the up-regulated genes include several involved in oxidative stress tolerance responses, although the factors that trigger such alterations remain unknown. In a tri-species *in vitro* model including *S. mutans, C. albicans* and probiotic *Lactobacillus *, Zeng *et al.* recently found that of the probiotic *Lactobacillus* strains tested, *L. plantarum* demonstrated superior inhibition on the growth of *C. albicans* and *S. mutans* and disruption of virulent biofilm formation with reduced bacterial cell and EPS components [90]. Inclusion of *L. plantarum* in the community was shown to disrupt the *S. mutans* and *C. albicans* cross-kingdom interactions and cause down expression of genes involved in the aforementioned processes including carbohydrate metabolism and EPS production in both *S. mutans* and *C. albicans* and resistance to antifungal medication in *C. albicans*. In addition, *Lactobacillus* genes for production of antimicrobial peptide plantaricin were also significantly upregulated. Interestingly, such anti-microbial effects were measured only when the community was grown in high-sucrose (1%) conditions but not in low-sucrose (0.1%) conditions. Acid production from sugar fermentation and the pH dependent antimicrobial activity of plantaricin were thought to be part of the contributing factors, since plantaricin is most active at pH 5.0.

Several *Lactobacillus* sp. were found to produce hydrogen peroxide, although relative to some other oral bacteria, such as *S. sanguinis* and *S. gordonii* [92], the level of hydrogen peroxide by some of the *Lactobacillus* sp. such as *L. fermentum* was found to be extremely low under the conditions studied. Like *S. sanguinis* and *S. gordonii* [92], hydrogen peroxide production by *Lactobacillus* sp. also appears to be regulated in response to environmental conditions including availability of fermentable sugars [93, 94] (Huang and Wen, personal communication) and probably the presence of other different bacterial species [36]. Currently, little information is available concerning the genetics and genetic regulation of hydrogen peroxide production in lactobacilli.

*L. fermentum* and several other *Lactobacillus* sp. were recently reported to possess nitric oxide synthase activity which utilizes l-arginine to produce nitric oxide [95, 96]. In addition, some *L. fermentum* sp. were also shown to have nitrite reductase that utilizes nitrite to generate nitric oxide [96]. A reactive nitrogen species, nitric oxide can react with superoxide anion (O_2_^-^) forming a potent oxidant peroxynitrite (ONOO^-^), which is a strong oxidant with a broad spectrum of antimicrobial activity [97, 98]. However, no nitric oxide synthase gene has been identified in any of these *Lactobacillus* strains, and it is currently unknown if nitric oxide production is species-specific.

It is becoming clear that while many *Lactobacillus* sp. produce various antimicrobial activities, the efficacy differs between different species and under different environmental conditions [78, 82, 90, 93]. How production of hydrogen peroxide, nitric oxide and/or bacteriocins in *L. casei* and other *Lactobacillus* sp. is regulated in the complex plaque microbiota and what impact these antimicrobials possess on the microecology and ultimately oral health await further investigation.

## Summary

*Lactobacillus* sp. are major contributors to dental caries, especially the development of advanced caries lesions in both adults and children. Unlike their cariogenic partner mutans streptococci, *Lactobacillus* sp. alone does not adhere the tooth surface efficiently. However, in the presence of *S. mutans* and some other primary colonizers, their ability to establish on a tooth surface can be significantly enhanced, although differences exist between the different species. Mechanical retention, including trapping in food remnants and plaque biofilms and especially the glucan scaffold of *S. mutans,* is a major factor contributing to *Lactobacillus* establishment. Besides, adhesin-receptor mediated active intercellular contact and probably metabolites associated interactions are also involved in the *S. mutans-*facilitated *Lactobacillus* adherence and persistence. On the other hand, *Lactobacillus* sp. also produce various broad-spectrum antimicrobials, including weak acids and bacteriocins. There is evidence that production of the antagonistic factors is regulated in response to environmental cues including presence of other bacterial species. It is hypothesized that as the lactobacilli grow to certain degree, such antagonistic activities become detrimental to the community, especially those in close proximity like *S. mutans *, which in turn weakens their own ability to maintain at the sites. Thus, the inter-species interaction between *S. mutans* and *Lactobacillus* sp. is a double-edged sword in terms of the development of plaque microbiota. If it is true, it also explains why neither *S. mutans* nor *Lactobacillus* is always present at caries sites [4, 97, 99]. Further studies will be needed to better understand the molecular mechanisms that govern the interactions between *S. mutans* and major species of *Lactobacillus* and how environmental conditions influence the inter-species interactions and ultimately, oral health and disease.

## Supplementary Data

Supplementary material 1
