# Supplementary material for: Lactobacilli and human dental caries: more than mechanical retention
Source: Microbiology (Reading). 2022 Jun 7;168(6):001196. doi: 10.1099/mic.0.001196 (PMC10233465; doi:10.1099/mic.0.001196)
Supplement: Supplementary material 1 [file mic-168-1196-s001.pdf]

Lactobacilli and Human Dental Caries: More Than Mechanical Retention

Zezhang T. Wen, Xiaochang Huang, Kassapa Ellepola, Sumei Liao, and Yihong Li

Supplemental Table: Major characteristics of selected *Lactobacillus* spp.

	L. casei/paracasei	*L. fermentum*	*L. rhamnosus*	*L. gasseri*	*L. salivarius*	*L. plantarum*
**Major location (1, 2)**	Oral mucosa, plaque, carious lesions, saliva	Plaque, caries lesions, saliva, and top of the tongue	Oral mucosa, top of the tongue, saliva, plaque, and carious lesions	Plaque, carious lesions, and saliva	Oral mucosa, saliva; top of the tongue, some plaque and carious lesions,	Oral mucosa, top of the tongue, and some plaque and carious lesions
**Sugar fermentation pattern**	Mainly facultatively heterofermentative	Obligately heterofermentative	Facultatively heterofermentative	Homofermentative	Homofermentative	Homofermentative
**Sugars fermented** (3-5)						
Amygdalin	+		+	+	-	+
Arabinose	-	+	+			+
Cellobiose	+	+	+	+	-	+
Esculin	+	-	+			+
Gluconate	+		+			+
Galactose		+		+	+	
Lactose				+	+	
Mannose		+		-	-	
Mannitol	+		+	+	+	+
Melezitose	+	-	+			+
Melibiose	-	+	-	+	+	+
Raffinose	-	+	-	+	+	+
Ribose	+	+	+			+
Sorbitol	+		+			+
Salicin				+	-	
Sucrose	+	+	+	+	+	+
Trehalose		+		+	+	
Xylose	-	+	-			+
Xylitol						
**Oligosaccharides utilized** (6, 7)						
Fructans	+					+
Levans	+					+
Inulin	+					
Fructooligosaccharides	+		+			+
Starch	+	+				+
Dextran	+	+				+
Galactosyllactose	+	+	+			+
Xylooligosaccharides		+				
Maltose/maltodextrin	+	+	+	+	+	+

Human milk oligosaccharides	+		+	+		+
**Collagen binding proteins (1, 8)**	+	+	+	+	+	+
**Fibronectin binding** (8)	+					+
**Mucin binding** (8)	+	+				+
**S-layer** (9)	-	-		+		
**Acid tolerance response**	Increases in both the activity and amount of F_0F_1- ATPase, a pH-inducible DNA repair system, maintenance of pH homeostasis and alterations in cell envelope, arginine deiminase pathway, and amino acid utilization (10)	Arginine deiminase system involved in regulating intracellular pH (11)	The F_1F_0 ATPase and stress proteins (12)	GroEL, GroES, DnaK, DnaJ, and Clp protease chaperones play a role in protection against intracellular aggregation of proteins during stress (10)	F_0F_1- ATPase, Production of urease (13)	H^+-ATPase proton pump intracellular NAD^+/NADH, arginine deiminase system, glutamate decarboxylase (GAD) system (14) and malolactic acid fermentation (13, 15)
**Biofilm formation**	Very limited capacity on polystyrene, glass and hydroxylapatite surfaces (16)	Limited biofilms on polystyrene and glass surfaces (17)	Poor biofilms on polystyrene and glass surfaces (17)	Very limited biofilms on polystyrene and glass surfaces (17)	Limited biofilms on polystyrene and glass surfaces (17)	Poor biofilms on hydroxylapatitic surface (18)
**Biofilm formation when co-cultivated with *S. mutans, Actinomyces* sp., *C. albicans***	Enhanced by ~2-logs when co-cultivated with *S. mutans* under both static and continuous flowing conditions; and adhesins P1 and GtfB are involved (16, 17)	Reduced when grown together with *S. mutans* by >1-log; *L. fermentum* also significantly reduced *S. mutans* biofilms (17)	Increased by as much as 2-logs when co-cultivated with *S. mutans* (17)	Increased by ~2-logs when co-cultivated with *S. mutans* (17)	Showed no major difference when co-cultivated with *S. mutans* (17); but *L. salivarius* also inhibited *S. mutans* in mixed-species model (19)	Enhanced by *Actinomyces* sp. and *S. mutans* by 4-7-fold (18); but *L. plantarum* also significantly inhibited *S. mutans* and *C. albicans* in a mixed-species model at 1% sucrose, but not at 0.1% sucrose (19)
**Number of PTS systems** (4, 5)<br>Strain, complete (incomplete) PTS systems	ATCC 334, 17 (14)	SNUV175, 3(6) *	1.0320, 33(18) *	ATCC 33323, 15(10)	UCC118, 7(3)	WCFS1, 25(13)
**Hydrogen peroxide production**	-	+ (20)		+ *	+ *	+ (21)
**Bacteriocin production** [#]	+	+	+	+	+	+

Notes: PTS, phosphoenolpyruvate-dependent phosphotransferase system; +, most strains tested are positive; -, most strains tested are negative; #, One or more bacteriocins have been reported in the strains studied. *, Huang and Wen, personal communication.

REFERENCES

1. Caufield PW, Schon CN, Saraithong P, Li Y, Argimon S. Oral lactobacilli and dental caries: a model for niche adaptation in humans. J Dent Res. 2015;94(9 Suppl):110S-8S.
2. Badet C, Thebaud NB. Ecology of lactobacilli in the oral cavity: a review of literature. Open Microbiol J. 2008;2:38-48.

3. Hammes WP, Vogel RF. The genus Lactobacillus. In: Wood BJB, Holzapfel WH, editors. The Genera of Lactic Acid Bacteria. 2: Blackie Academic and Professional; 1995. p. 19-54.
4. Verce M, De Vuyst L, Weckx S. Comparative genomics of *Lactobacillus fermentum* suggests a free-living lifestyle of this lactic acid bacterial species. Food microbiology. 2020;89:103448.
5. Francl AL, Thongaram T, Miller MJ. The PTS transporters of *Lactobacillus gasseri* ATCC 33323. BMC Microbiol. 2010;10:77.
6. Zunga M, Yebra MJ, Monedero V. Complex Oligosaccharide Utilization Pathways in Lactobacillus. Curr Issues Mol Biol. 2021;40:49-80.
7. Nadkarni MA, Deshpande NP, Wilkins MR, Hunter N. Intra-species variation within *Lactobacillus rhamnosus* correlates to beneficial or harmful outcomes: lessons from the oral cavity. BMC genomics. 2020;21(1):661.
8. Muscariello L, De Siena B, Marasco R. Lactobacillus cell surface proteins involved in interaction with mucus and extracellular matrix components. Current microbiology. 2020;77(12):3831-41.
9. Hynonen U, Palva A. Lactobacillus surface layer proteins: structure, function and applications. Applied microbiology and biotechnology. 2013;97(12):5225-43.
10. Azcarate-Peril MA, Altermann E, Goh YJ, Tallon R, Sanozky-Dawes RB, Pfeiler EA, et al. Analysis of the genome sequence of *Lactobacillus gasseri* ATCC 33323 reveals the molecular basis of an autochthonous intestinal organism. Appl Environ Microbiol. 2008;74(15):4610-25.
11. De Angelis M, Mariotti L, Rossi J, Servili M, Fox PF, Rollán G, et al. Arginine catabolism by sourdough lactic acid bacteria: purification and characterization of the arginine deiminase pathway enzymes from *Lactobacillus sanfranciscensis* CB1. Appl Environ Microbiol. 2002;68(12):6193-201.
12. Bang M, Oh S, Lim K-S, Kim Y, Oh S. The involvement of ATPase activity in the acid tolerance of *Lactobacillus rhamnosus* strain GG. International Journal of Dairy Technology. 2014;67(2):229-36.
13. Cotter PD, Hill C. Surviving the acid test: responses of Gram-positive bacteria to low pH. Microbiol Mol Biol Rev. 2003;67(3):429-53.
14. Guo Y, Tian X, Huang R, Tao X, Shah NP, Wei H, et al. A physiological comparative study of acid tolerance of *Lactobacillus plantarum* ZDY 2013 and *Lactobacillus plantarum* ATCC 8014 at membrane and cytoplasm levels. Annals of Microbiology. 2017;67(10):669-77.
15. Olsen EB, Russell JB, Henick-Kling T. Electrogenic L-malate transport by *Lactobacillus plantarum*: a basis for energy derivation from malolactic fermentation. J Bacteriol. 1991;173(19):6199-206.
16. Wen ZT, Yates D, Ahn SJ, Burne RA. Biofilm formation and virulence expression by *Streptococcus mutans* are altered when grown in dual-species model. BMC Microbiol. 2010;10:111.
17. Wen ZT, Liao S, Bitoun JP, De A, Jorgensen A, Feng S, et al. *Streptococcus mutans* displays altered stress responses while enhancing biofilm formation by *Lactobacillus casei* in mixed-species consortium. Front Cell Infect Microbiol. 2017;7:524.
18. Filoche SK, Anderson SA, Sissons CH. Biofilm growth of *Lactobacillus* species is promoted by *Actinomyces* species and *Streptococcus mutans*. Oral Microbiol Immunol. 2004;19(5):322-6.
19. Zeng Y, Fadaak A, Alomeir N, Wu TT, Rustchenko E, Qing S, et al. *Lactobacillus plantarum* disrupts *S. mutans-C. albicans* cross-kingdom biofilms. Front Cell Infect Microbiol. 2022;12:872012.
20. Huang X, Friel KL, Waters JP, Urbina DM, Elepola KJ, Li Y, et al. Analysis of cariogenic potential of lactobacillus isolates from patients with severe early childhood caries. The 97th General Session of the IADR Washington, DC2020.
21. Lorquet F, Goffin P, Muscariello L, Baudry JB, Ladero V, Sacco M, et al. Characterization and functional analysis of the poxB gene, which encodes pyruvate oxidase in *Lactobacillus plantarum*. J Bacteriol. 2004;186(12):3749-59.
